# Red Sea Atlantis II brine pool nitrilase with unique thermostability profile and heavy metal tolerance

**DOI:** 10.1186/s12896-016-0244-2

**Published:** 2016-02-11

**Authors:** Sarah A. Sonbol, Ari J. S. Ferreira, Rania Siam

**Affiliations:** Biology Department and YJ-Science and Technology Research Center, American University in Cairo, New Cairo, 11835 Egypt

**Keywords:** Nitrilase, Atlantis II Deep brine pool, Red Sea, Metagenomics, Heavy metals tolerance, Thermostability

## Abstract

**Background:**

Nitrilases, which hydrolyze nitriles in a one-step reaction into carboxylic acids and ammonia, gained increasing attention because of the abundance of nitrile compounds in nature and their use in fine chemicals and pharmaceutics. Extreme environments are potential habitats for the isolation and characterization of extremozymes including nitrilases with unique resistant properties. The Red Sea brine pools are characterized by multitude of extreme conditions. The Lower Convective Layer (LCL) of the Atlantis II Deep Brine Pool in the Red Sea is characterized by elevated temperature (68 °C), high salt concentrations (250 ‰), anoxic conditions and high heavy metal concentrations.

**Results:**

We identified and isolated a nitrilase from the Atlantis II Deep Brine Pool in the Red Sea LCL. The isolated 338 amino-acid nitrilase (NitraS-ATII) is part of a highly conserved operon in different bacterial phyla with indiscernible function. The enzyme was cloned, expressed and purified. Characterization of the purified NitraS-ATII revealed its selectivity towards dinitriles, which suggests a possible industrial application in the synthesis of cyanocarboxylic acids. Moreover, NitraS-ATII showed higher thermal stability compared to a closely related nitrilase, in addition to its observed tolerance towards high concentrations of selected heavy metals.

**Conclusion:**

This enzyme sheds light on evolution of microbes in the Atlantis II Deep LCL to adapt to the diverse extreme environment and can prove to be valuable in bioremediation processes.

**Electronic supplementary material:**

The online version of this article (doi:10.1186/s12896-016-0244-2) contains supplementary material, which is available to authorized users.

## Background

Nitriles are organic compounds that contain a cyano (CN) functional group. These compounds are abundant in nature, produced by selected plants, animals [1], bacteria, fungi and algae [2]. Nitriles can also be byproducts of industrial processes or valuable industrial products [1]. Processing of nitriles can occur either chemically or enzymatically. To this end, nitrilases (EC 3.5.5.1) are one of the most important enzymes as they hydrolyze nitriles (R-CN) directly into their corresponding carboxylic acids (R-COOH) and ammonia (NH_3_) [1, 3, 4]. Nitrilases are a subgroup of the carbon-nitrogen (C-N) hydrolase superfamily. Members of this superfamily are classified into 13 subgroups and are involved in non-peptide C-N hydrolysis [5]. This superfamily was first classified by Brenner (2002) as the nitrilase superfamily, even though one branch only exhibited true nitrilase function [1]. All members of the C-N hydrolase superfamily are characterized by the presence of a catalytic triad of glutamate-lysine-cysteine residues and an α-β-β-α sandwich fold [5]. According to their substrate specificity, nitrilases are classified into three major classes: aromatic, aliphatic and arylacetonitrile nitrilases. Nevertheless, some nitrilases have shown broad substrate specificity [1, 3].

As nitrile compounds are extensively used in several industries and in the synthesis of a variety of fine chemicals and pharmaceutical compounds [1], interest towards the use of nitrilases has risen as an alternative to conventional chemical methods. Unlike those, nitrilases require milder reaction conditions without the production of toxic byproducts [1, 6–8]. Furthermore, nitrilases can be stereo- and/or regio-selective, allowing the production of specific isomers. Additionally, some nitrilases can hydrolyze a single cyano group in dinitriles or polynitriles producing cyanocarboxylic acids, which are used in different industries [1, 8]. Nitrilases were successfully utilized in manufacturing different types of polymers, such as nylon-6, and other chemicals such as nicotinic and acrylic acids. They were also used in pharmaceutical industries including the manufacturing of (*S*)-ibuprofen, a widely used non-steroidal anti-inflammatory drug [6]. Moreover, nitrilases were found to be of extreme importance in bioremediation as in the detoxification of cyanide containing wastes and in nitrile herbicides degradation [1, 3].

The presence of nitrilases is infrequent in nature. Nonetheless, their frequency is considered high in the bacterial kingdom when compared to plants and fungi [1]. However, the role of nitrilases in bacterial metabolism is still unclear. Generally nitrilases are inducible enzymes [1, 9] and may play roles in complex pathways for the detoxification of xenobiotics or cyanogenic compounds [1, 6, 10]. Interestingly, some bacterial strains that possess nitrilases were found to be able to utilize nitriles as a sole source of carbon and nitrogen [1, 6].

Isolation and characterization of nitrilases were performed by conventional screening methods through culturing of microorganisms on selective media with nitrile substrates as sole carbon or nitrogen sources; thus, allowing the growth of nitrilase-producing microorganisms only from which nitrilases can then be isolated [11]. Despite such tedious and time-consuming approach, different nitrilases were isolated, identified and characterized using this strategy. However, since cultured microorganisms represent less than 0.1 % of the microbial world [5], the trend towards studying the unfathomable world of uncultured microorganisms seems promising. Metagenomic studies have provided us with a plethora of microbial genomic sequences from which novel and diverse enzymes could be characterized. Extracting microbial genomes from extreme environments increases the possibility of the identification of extremozymes and understand adaption of microbes to these environments [11].

Studies on the Red Sea revealed the presence of about 25 brine pools in its deepness [12]. Those are geothermal salt-enriched underwater lakes that are found in depressions in the seafloor of the central and northern Red Sea [13]. The high density of brine water stabilizes the pool and isolates it from the above water [14]. The largest brine pool in the Red Sea is Atlantis II Deep (ATII). It is at a maximum depth of 2194 m, located near the central rift of the Red Sea between Saudi Arabia and Sudan [14]. This brine pool is a unique ecosystem characterized by elevated temperatures and salinity [12]. Difference in temperature, salinity and oxygen content segregates the ATII into four brine layers. The Lower Convective Layer (LCL), the deepest of the four ATII layers, has the harshest condition; highest temperature (68 °C), salinity (250 parts per thousand or 7.5 times that of the normal sea water) [12, 13] and heavy metal content, in addition to a pH value of 5.3. Recently, genes coding for a mercuric reductase and a lipase were cloned from environmental DNA isolated from the ATII LCL and the respective proteins were heterologously expressed and biochemically characterized, unveiling their adaptations to function under such extreme conditions [15, 16] . In this study, a nitrilase was identified from the LCL metagenomic dataset. The Atlantis II nitrilase (NitraS-ATII) gene was isolated, expressed and characterized. The expressed enzyme (NitraS-ATII) was selective towards dinitriles. Heavy metal tolerance and thermostability were compared to that of *Rhodobacter sphaeroides* LHS-305 nitrilase, a highly similar nitrilase isolated and biochemically characterized by Wang et al. [17]. Our results have shown that NitraS-ATII has higher thermal stability than *R. sphaeroides* LHS-305 nitrilase and is highly tolerant to different heavy metals.

## Methods

### Sampling, DNA extraction and sequencing

During the KAUST/WHOI/HCMR spring 2010 expedition onboard the R/V Aegeo, water samples were obtained from the LCL Atlantis II Deep layer (21° 20.72’ N and 38° 04.59’ E) by means of Niskin bottles mounted on a *rosette* supplied with a CTD (Conductivity, Temperature and Depth) meter. Samples were sequentially filtered through mixed cellulose ester filters (nitrocellulose / cellulose acetate) of pore sizes 3.0 μm, 0.8 μm and 0.1 μm (Millipore),which were immediately stored in sucrose buffer at −20 °C until being further processed. Prokaryotic DNA was extracted from cells retained on the 0.1 μm filter, using the Marine DNA Isolation Kit (Epicenter, USA) according to the manufacturer’s recommendations. The concentration of the extracted DNA was determined using a NanoDrop 3300 Fluorospectrometer (Thermo Scientific, USA) and a Quant-iT™ PicoGreen® dsDNA Kit (Invitrogen, USA). Extracted DNA was sequenced using the shotgun sequencing approach on a GS FLX Titanium pyrosequencer (454 Life Sciences), creating a dataset with about 8 million reads for the Atlantis II Deep LCL. An additional dataset of assembled reads (ATBRLCL01A1_assembly) was created using Newbler assembler v2.6 giving rise to 28,462 contigs (larger than 100 bp) with an average contig size of 1912 bp. ORFs within these contigs were identified and annotated using MetaGeneAnnotator [18, 19].

### Computational analyses

The Pfam signature for the Carbon-Nitrogen hydrolase functional domain (PF00795) was obtained from Pfam 26.0 [20] and used to extract reads with the corresponding domain from the LCL dataset using HMM scan [21]. The extracted reads were assembled and visualized using the Phred-Phrap-Consed tool [22–26] giving rise to a number of contigs. The contig with the greatest number of reads was subjected to a BLASTn [27] search against the ATBRLCL01A1_assembly dataset to determine a larger genomic region containing the Pfam signature. Within this genomic region, ORFs close to the Pfam signature were identified and manually annotated in the Artemis platform (release 13.2.0 [28]). Promoter regions were then identified using the bprom tool [29] and Shine-Dalgarno sequences were annotated manually. The sequence of the ORF of interest was compared, using the BLASTx algorithm [27], to the non-redundant protein database (nr) of the National Center for Biotechnology Information (NCBI, USA) for identification of a possible new nitrilase gene. To ensure the presence and correct position of the catalytic triad of nitrilases in the computationally identified protein, multiple protein sequence alignment of the translated gene to close homologs was performed using ClustalW2 [30, 31].

The Phyre2 tool [32] was used for homology modeling of NitraS-ATII and *R. sphaeroides* nitrilase, a highly similar nitrilase (GenBank accession number JN635494) [17]. Three-dimensional structures were visualized and superimposed using PyMOL™ Evaluation Product© (DeLano Scientific LLC, 2008). For determination of salt bridges in NitraS-ATII and *R. sphaeroides*nitrilase, ESBRI online tool [33–36] was utilized.

### Amplification and sequencing of the NitraS-ATII CDS and its upstream regulatory elements

Whole genome amplification of the metagenome was done using REPLI-g® Mini Kit (Qiagen) according to the manufacturer’s specifications. A one-time whole-genome amplified LCL metagenomic DNA sample was used as template to amplify the NitraS-ATII gene and adjacent regulatory elements. Different sets of primers, designed using Primer3Plus software [37] based on the consensus sequence of contig00026 of ATBRLCL01A1_assembly, were used for this purpose (Additional file 1: Figure S1 and Additional file 1: Table S2). The amplified sequences were then extracted from agarose gel using QIAquick® gel extraction kit (Qiagen) followed by amplicon TA cloning into pGEM®-T Easy cloning vector (Promega). The recombinant plasmids were transformed into *E. coli* Top ten strain, grown on LB agar containing 0.5 mmol.L^-1^ IPTG, 40 μg.mL^-1^ X-gal and 100 μg.mL^-1^ ampicillin. Colony PCR was performed for several positive white clones using the same primers used in amplification to confirm the presence of the amplified sequences. Plasmids were extracted from cultures of colonies with positive colony PCR results using PureYield™ Plasmid Miniprep System (Promega). Direct sequencing, or sequencing of generated recombinant plasmids, was performed on an ABI 3730xl DNA Analyzer using one of the amplification primers, the T7 forward or the Sp6 reverse primers and the BigDye® Terminator v3.1 Cycle Sequencing Kit (Applied Biosystems®). Sequencing results were visualized using BioEdit Sequence Alignment Editor (version 7.1.3.0) [38].

### Expression of NitraS-ATII in *E. coli* BL21 (DE3)

For recombinant expression into *E. coli* BL21 (DE3), a synthesized gene - based on the sequence amplified from the metagenomic DNA - with optimized codons for *E. coli* expression was obtained from GenScript in pET-28b + with a C-terminal His-tag. The NitraS-ATII ORF was designed to be flanked by *Sac*I and *Hind*III restriction sites. The obtained recombinant plasmid was designated as p-NitraS-ATII.

p-NitraS-ATII was initially transformed into *E. coli* Top10 by electroporation. Transformed cells were grown on LB agar plates with kanamycin (50 μg.mL^-1^). Plasmids were extracted from cultures using PureYield™ Plasmid Miniprep System (Promega) followed by their transformation into *E. coli* BL21 (DE3) for expression and sequencing. An overnight culture of the transformed cells was subcultured in LB broth with kanamycin (50 μg.mL^-1^). Cultures were allowed to grow at 37 °C and 225 rpm to an OD_600_ of ~0.6 at which point induction of expression was performed for 2 h at 37 °C using 0.1 mmol.L^-1^ IPTG. Pellets from 25 and 50 mL cultures were preserved at−80 °C for analysis. All cell lysates were analyzed by SDS-PAGE on 12 % gels, which were subsequently stained with Coomassie blue R250 according to the method of Laemmli [39].

### Purification of His-tagged NitraS-ATII by Ni^2+^ column

Cell pellets were treated as described by Kim et al. [7] and Yeom et al. [4] with some modifications. Briefly, pellets of induced cells were subjected to freezing (in ice cold ethanol) and thawing (42 °C), followed by re-suspension in binding buffer, pH 8.0 (20 mmol.L^-1^Na_2_HPO_4_, 40 mmol.L^-1^ imidazole and 500 mmol.L^-1^ NaCl). Lysozyme (1.0 mg.mL^-1^) and phenylmethylsulfonyl fluoride (PMSF, 1.0 mmol.L^-1^) were added and the suspension was incubated on ice for 30 min with occasional shaking. Cells were subjected to sonication with 10 s bursts interrupted by 10 s intervals for a total period of 10 min, followed by centrifugation to separate the supernatant from the cell debris. The protein (NitraS-ATII) was purified using Ni^2+^ affinity chromatography with Ni-NTA agarose resin (Invitrogen™). Purification was done according to manufacturer’s native condition specifications at 4 °C. The His-tagged NitraS-ATII was eluted with 20 mmol.L^-1^ Na_2_HPO_4_ buffer (pH 8.0) containing 500 mmol.L^-1^ imidazole, 500 mmol.L^-1^ NaCl and 50 % glycerol. Coomassie-stained 12 % SDS-PAGE gels were used to visualize the protein during each purification stage according to the method of Laemmli [39]. Protein concentration was determined using the Pierce™ BCA Protein Assay Kit (Thermo Scientific). A standard curve was obtained using known concentrations of standard Bovine Serum Albumin (BSA), and colorimetric measurements were obtained at λ = 595 nm using a FLUOstar OPTIMA microplate reader (BMG LABTECH).

### Nitrilase activity assay

A preliminary colorimetric activity assay, developed by Banerjee et al. [40] was performed with minor modifications. Harvested cells from 25 mL overnight cultures of transformed cells (induced and uninduced) were washed with 10 mmol.L^-1^ phosphate buffer (pH 7.2), and suspended in the same buffer according to their OD_600_ (pellets from cultures with original OD_600_ ~ 1.0 were re-suspended in 1.0 mL buffer). In a 230,0 μL reaction, 6,00 μL of nitrile substrate (from an 1,0 mol.L^-1^ stock solution) and 23,0 μL of prepared cell suspension were added to 201,0 μL of phosphate buffer (10 mmol.L^-1^, pH 7.2) containing 0.01 % bromothymol blue. The reaction mixture was incubated at 50 °C for 6 h. A color change of the bromothymol blue indicator from bluish-green to yellow was considered a positive result for nitrilase due to liberation of acid into the media. As positive control for the assay, induced cells transformed with recombinant pET-28a + carrying a nitrilase gene from *Rhodococcus rhodochrous* ATCC 33278 [4] were used. Negative controls were non-transformed and uninduced cells.

For quantitative nitrilase activity assay, we used a spectrophotometric-based method modified from that developed by Goyal et al. [41] and from the fluorometric assay developed by Banerjee et al. [42]. The method is based on measuring the amount of liberated ammonia by the action of the nitrilase on its substrate. In a 100 μL reaction, 10 μL of purified protein (100 μg.mL^-1^) were added to 50 mmol.L^-1^ potassium phosphate buffer (pH 7.0), 2 mmol.L^-1^dithiothreitol (DTT) and 400 mmol.L^-1^ substrate. The reaction was kept at 40 °C and 100 rpm for 30 min, and then stopped with an equal volume of 100 mmol.L^-1^ HCl. The aborted reaction mixture was centrifuged at 5000 x g for 10 min. To detect liberated ammonia, 10 μL of the reaction mixture were added to 140 μL of buffered alcoholic *o*-phthaldialdehyde / β-mercaptoethanol reagent. The isoindole derivative was allowed to develop for 30 min at 30 °C and 100 rpm. The final color intensity was measured at λ = 405 nm using the FLUOstar OPTIMA microplate reader (BMG LABTECH).

The reagent used in the assay was prepared 1 day before the assay as described by Banerjee et al. [42]. Stock solutions of alcoholic *o*-phthaldialdehyde, 100 mg *o*-phthaldialdehyde dissolved in 10 mL absolute ethanol, and of alcoholic β-mercaptoethanol, 50 μl β-mercaptoethanol in 10 mL absolute ethanol, were initially prepared. For preparing the working reagent, 2.25 mL of both alcoholic *o*-phthaldialdehyde and alcoholic β-mercaptoethanol were added to 45.5 mL of 200 mmol.L^-1^ potassium phosphate buffer (pH 7.4). A standard curve was created using NH_4_Cl for determination of the ammonia concentration. The specific activity of the enzyme was measured in U/mg of total protein. One unit (U) of nitrilase activity was defined as the amount of ammonia in micromoles produced in 1.0 min by the enzyme (μmoL.min^-1^) under the previously mentioned conditions.

### Substrate specificity

For determination of substrate specificity, several nitriles belonging to different classes were tested: acetonitrile (simple aliphatic nitrile), glutaronitrile, succinonitrile (aliphatic dinitriles), mandelonitrile (arylacetonitrile) and benzonitrile (aromatic nitrile) (Sigma-Aldrich). In addition, acetamide and benzamide (Sigma-Aldrich) were used for the detection of possible amidase activity.

### Effect of pH on NitraS-ATII activity

NitraS-ATII activity was assessed at different pHs, ranging from 3.5 to 11. Assays were developed as previously described using succinonitrile as a substrate and acetate buffer (pH 3.5-5.0), phosphate buffer (pH 6.0–8.0) or carbonate buffer (pH 9.0–11.0).

### Enzyme kinetics

NitraS-ATII kinetics were studied using succinonitrile as substrate. Initial velocities were determined in 10 min reactions with fixed concentration of purified NitraS-ATII (100 μg.mL^-1^) and different concentrations of succinonitrile (0–975 mmol.L^-1^). The Michaelis-Menten kinetic parameters (K_M_, v_max_ and k_cat_) were determined with the aid of GraphPad Prism®(Windows® version 5.00).

### Enzyme thermal stability and thermosensitivity

For determination of the enzyme thermal stability, purified NitraS-ATII was incubated for different time intervals (30 s to 60 min) at different temperatures (0–80 °C) before assaying the residual activity by means of the previously mentioned quantitative assay. The residual activities of NitraS-ATII after 30 s and 1 min incubations at different temperatures were compared to those of *Rhodobacter sphaeroides* LHS-305 nitrilase, subjected to the same treatment. The thermosensitivity of NitraS-ATII was assayed at different temperatures (15–70 °C) under aforementioned conditions and was compared to *R. sphaeroides* LHS-305 nitrilase thermosensitivity profile.

### Effect of salt on the enzyme activity

For examination of the salt tolerance for NitraS-ATII, the nitrilase activity was assessed at different NaCl concentrations (0–4.0 mol.L^-1^) under the previously described conditions.

### Effect of different metals on NitraS-ATII activity

The nitrilase activity of NitraS-ATII was assessed in presence of different metals at different concentrations. Reaction conditions were the same as previously mentioned with the exception that no DTT was added to the reaction mixture since it is a known complexant of metal ions such as nickel, copper, zinc, cadmium [43] and mercury [44]. The reaction mixtures had concentrations of NiSO_4_, CdCl_2_, CoCl_2_, ZnCl_2_, MgSO_4_ or MnSO_4_ ranging from 1.0 to 25.0 mmol.L^-1^. In case of HgCl_2_, 6 and 12 μmol.L^-1^concentrations were used while 0.5, 1.0 and 2.0 mmol.L^-1^concentrations were used in case of CuSO_4_. Activities in the presence of these metal ions were compared to those of *R. sphaeroides* LHS-305 nitrilase subjected to the same conditions.

### Experimental data analysis

All experiments in this study were done at least in triplicates. Data points were presented as mean ± SE. Graphs were plotted using GraphPad Prism®(Windows® version 5.00).

## Results

### Identification of nitrilase sequences in the Atlantis II Brine LCL database

HMM scan over 8 million ATII-LCL 454 reads provided 179 reads that contained the CN_hydrolase functional domain signature (Pfam PF00795). Those reads were assembled forming 15 contigs (Additional file 1: Table S1). The one with the largest number of reads (contig15: 40 reads, 1209 bp) contained a putative CDS with the entire CN_hydrolase functional domain. Its consensus sequence aligned perfectly with contig00026 (66.091 kb) within ATBRLCL01A1_assembly dataset (from position 42,805 to position 44,011). Visualization of this region of contig00026 in Artemis [28] revealed that the CDS of interest was part of an operon of 9 ORFs (Additional file 1: Figure S2). Bprom [29] identified regulatory regions upstream of the first ORF in the operon:−10 and−35 boxes (sequences: CGCAATGAT and TTAAAG, respectively; Additional file 1: Figure S3 and Additional file 1: Table S3), and two binding sites for two transcriptional factors: purR, CGTTTTTT, and rpoD15, TTTTGTTT. ORFs in the operon were compared to NCBI non-redundant protein database (nr) using BLASTp [27] and subsequently annotated as follows: conserved hypothetical protein, putative nitrilase, putative radical SAM-domain containing protein, putative acetyltransferase, selenophosphate synthetase-related protein (AIR synthetase-related protein), conserved hypothetical protein, putative FAD-dependent oxidoreductase, conserved hypothetical protein and putative methylmalonyl-CoA mutase.

**Fig. 1:**
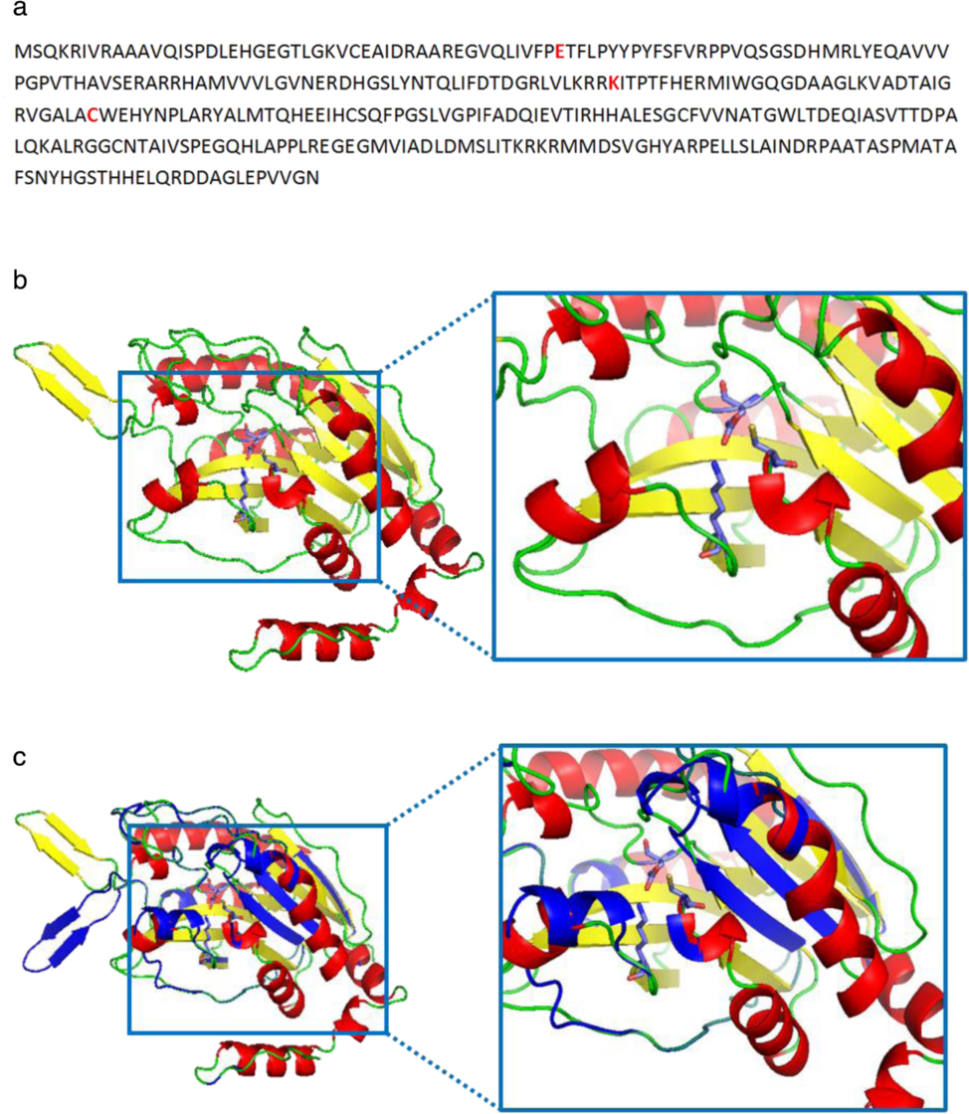
The NitraS-ATII protein. **a** Amino acid sequence of the native NitraS-ATII with the catalytic triad of glutamate (D47), lysine (K129) and cysteine (C163) shown in red. **b** Three dimensional model of NitraS-ATII protein. The model was obtained using Phyre2 tool with 100 % confidence and visualized using pyMOL. Secondary structures were colored as follows: α-helices in red, β-sheets in yellow and loops in green. Residues of the catalytic triad are shown as stick representation, with carbon atoms in cyan, nitrogen atoms in red, oxygen atoms in blue and sulfur atoms in yellow. **c** Superimposition of three-dimensional structure models of NitraS-ATII and nitrilase from *Rhodobacter sphaeroides* LHS-305. NitraS-ATII, colored according to secondary structure, was superimposed onto *R. sphaeroides* LHS-305 nitrilase, shown in blue. Residues of the catalytic triad showed perfect superimposition

The ORF containing the Pfam CN_hydrolase signature had several BLAST hits annotated as carbon-nitrogen hydrolases or aliphatic nitrilases (98 % identity to its best hit, carbon-nitrogen hydrolase from *Cupriavidus basilensis* OR16, GenBank accession number: EHP37962) and was consequentially named NitraS-ATII. However, BLAST hits with the highest identities to NitraS-ATII were annotated based on sequence similarities only, none having its activity experimentally tested. The encoded protein sequence of NitraS-ATII consisted of 338 amino acids. Functional domain search by Pfam [20] identified the conserved nitrilase catalytic triad; glutamate, lysine and cysteine at positions 47, 129 and 163, respectively (Fig. 1a). ClustalW2 [30, 31] revealed high conservation with several identified nitrilases (Additional file 1: Figure S4).

*Rhodobacter sphaeroides* LHS-305 nitrilase [17], showed 76 % identity and 84 % positives with NitraS-ATII. Since it had been cloned and biochemically characterized, we selected it as a control, for our study. Initially, 3D structure models of NitraS-ATII (Fig. 1b) and of *R. sphaeriodes*LHS-305 nitrilase were obtained with 100 % confidence using Phyre2 [32], using the crystal structure of nit6803 nitrilase (PDB c3wuyA) as a template [45], since it showed the highest identity (71 %) to both nitrilases. Upon superimposition of the 3D structure models for both nitrilases, few variations were observed; yet, the residues of the catalytic triad showed perfect superimposition (Fig. 1c). Analysis of the structure of NitraS-ATII and *R. sphaeriodes* LHS-305 nitrilase, using ESBRI, predicted 119 and 102 internal salt bridges, respectively.

### Expression and purification of NitraS-ATII

Based on the sequence amplified from the metagenomic DNA, a C-terminal His-tag synthesized gene with optimized codon usage was obtained from GenScript in pET-28b + (p-NitraS-ATII). A C-terminal His-tag was chosen to diminish the possibility of altered protein activity, since the active site was found to be close to the N-terminus. The recombinant plasmid was transformed into *E. coli* BL21 (DE3) for protein expression. Based on Expasy Compute pI/MW tool [46–48], the apparent MW of the induced protein was 38.83 kDa. The small increase in size when compared to the predicted native protein (37.12 kDa) was related to the extra histidine residues at the C-terminus and 2 extra methionine and glycine residues at the N-terminus, added to place the gene in frame with the expression vector (between *Sac*I and *Hind*III restriction sites).

**Fig. 2:**
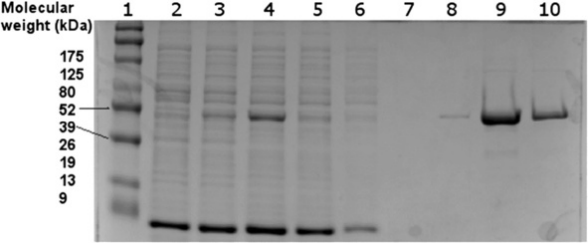
Purification of NitraS-ATII protein. Protein concentration and integrity at different purification steps were visualized on SDS-PAGE gel (12 %). *Lane 1*: ProSieve® Colour Protein Markers (Lonza), *lane 2*: non-transformed cells supernatant, *lane 3*: uninduced cells supernatant, *lane 4*: induced supernatant before purification, *lane 5*: NTA column flow-through, *lane 6*: first column wash, *lane7*: second column wash, *lane 8*: eluate fraction one, *lane 9:* eluate fraction two, *lane 10*: eluate fraction three

Optimization of heterologous expression conditions led to the highest amount of expressed protein after a 2 h induction period at 37 °C using 0.1 mmol.L^-1^ IPTG. Although this condition did not eliminate the presence of most of the expressed protein in the insoluble fraction, a considerable amount of soluble protein enabled purification under native conditions as described in the Materials and Methods section. Protein concentration and integrity were followed throughout the purification process by means of SDS-PAGE. Samples from different purification steps are shown on a 12 % SDS-PAGE gel in Fig. 2.

### Nitrilase activity and substrate specificity of NitraS-ATII

To assess the nitrilase activity of NitraS-ATII, a qualitative colorimetric assay using bromothymol blue following incubation of induced transformed cells for 6 h at 50 °C [40] was performed. To ensure the validity of this assay, the nitrilase activity of induced *E. coli* BL21(DE3) cells transformed with recombinant pET28a + carrying *Rhodococcus rhodochrous* ATCC 33278 nitrilase gene [4] was similarly assayed using the substrates acetonitrile and mandelonitrile. Nitrilase activity was assessed using untransformed *E. coli* BL21 (DE3) cultures, induced and uninduced p-NitraS-ATII cultures. Untransformed cells yielded a dark green color of the indicator, while transformed cells – induced and uninduced – showed yellowish coloration, indicating basal level expression of the recombinant protein and leakage of T7*lac* promoter, even though a drop in the pH was more prominent with the induced cells.

Negative results were obtained with acetonitrile, mandelonitrile and benzonitrile (data not shown), whereas positive results were observed with glutaronitrile and succinonitrile. However, with succinonitrile rather than glutaronitrile, a prominent difference in color between transformed and untransformed cells was observed (Additional file 1: Figure S5). Based on these results, the specificity of NitraS-ATII was determined to be towards aliphatic dinitriles.

In the quantitative spectrophotometric assay, DTT (2.0 mmol.L^-1^) was added to the reaction mixture to ensure that the cysteine residue in the enzyme active site was in its reducing form, since we observed lower NitraS-ATII activity in absence of a reducing agent (data not shown). Positive results were only obtained with glutaronitrile and succinonitrile, confirming our previous results of the dinitrile specificity of NitraS-ATII. Activity towards glutaronitrile was ~8.5 % of that towards succinonitrile, indicating the last as a preferred substrate. Additionally, we did not observe any amidase activity when acetamide and benzamide were used as substrates.

### Biochemical characterization of NitraS-ATII enzymatic activity

**Fig. 3:**
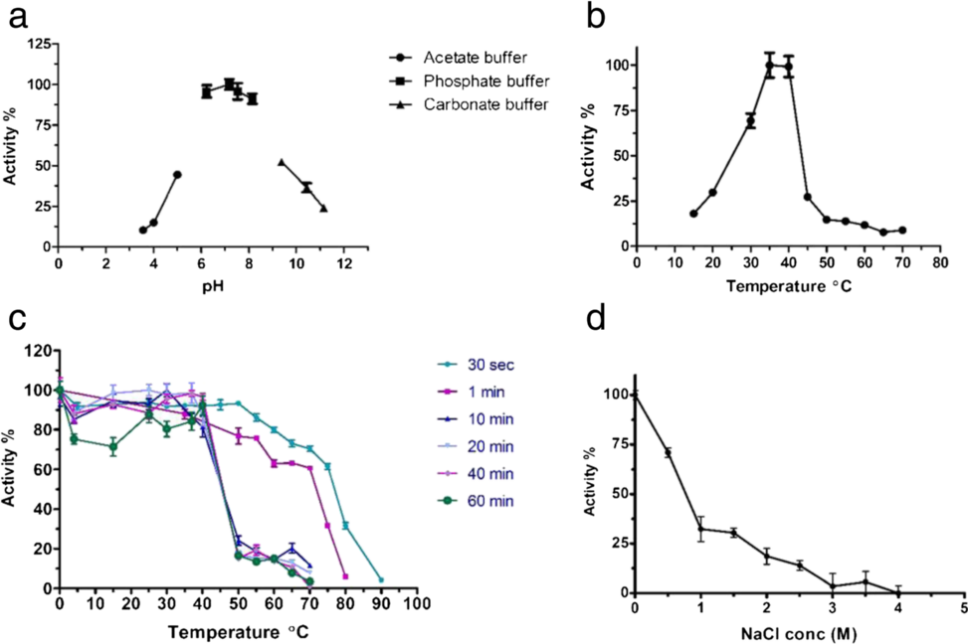
Biochemical characterization of NitraS-ATII. **a**. Effect of pH on nitrilase activity was assessed in acetate buffer (pH 3.5–5.0), phosphate buffer (pH 6.0–8.0) and carbonate buffer (pH 9.0–11.0). Optimum activity was achieved at pH 7.0. **b**. Effect of temperature on nitrilase activity. Optimum temperature was determined to be 40 °C and activity was nearly abolished at higher temperatures. **c**. Residual nitrilase activity after incubation at different temperatures for different periods. **d**. Nitrilase activity at different NaCl concentrations. Decrease in activity was observed with increase in salt concentration. All assays used 400 mmol.L^-1^ succinonitrile as substrate

The reaction between NitraS-ATII and succinonitrile was set using different buffers to test the enzymatic activity at different pHs. Surprisingly, the highest activity was achieved at pH 7.0 (Fig. 3a) and the best range for measuring this enzyme activity was identified to be pH 6.0–8.0. The enzyme lost its activity at low pHs and retained minimal activity at basic pHs.

**Fig. 4:**
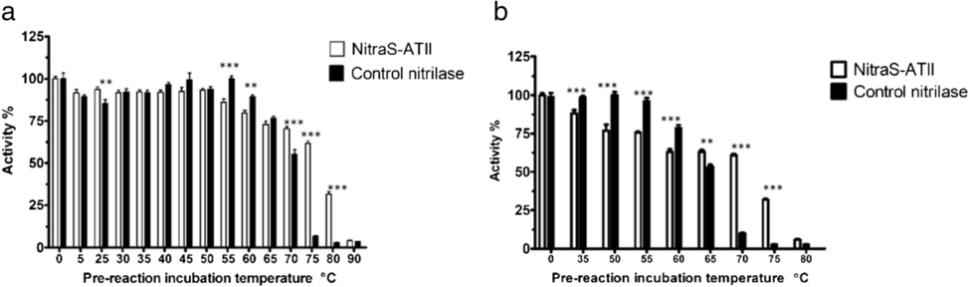
Thermal stability of NitraS-ATII. The enzymes were incubated at high temperatures for 30 s (**a**) or 1 min (**b**) prior to performing the reaction and measuring the residual activity. Two-way ANOVA test followed by Bonferroni post-hoc test was performed using GraphPad Prism®(Windows® version 5.00). *** and ** indicate *p*-values lower than 0.001 and 0.01, respectively

The optimum temperature for the reaction was 35–40 °C, and a sharp decrease in activity was observed at temperatures higher than 40 °C (Fig. 3b). For the *R. sphaeriodes* LHS-305 nitrilase, used as control, the optimum reaction temperature was 50 °C. Upon incubating NitraS-ATII at different temperatures for different periods of time (10–60 min) before starting the reaction, the residual activity of the enzyme decreased sharply at temperatures higher than 40 °C (Fig. 3c). However, we observed a significant difference between the residual activity of NitraS-ATII and *R. sphaeriodes* LHS-305 nitrilase, upon incubation at 70, 75 and 80 °C for 30 s or 1 min before initiating the reaction. For instance, NitraS-ATII retained 61.6 and 31.8 % of its activity after incubation at 75 °C for 30 s and 1 min, respectively. On the other hand, *R. sphaeriodes* LHS-305 nitrilase maintained only 6.8 and 2.8 % of its activity under the same conditions. Moreover, NitraS-ATII retained 70.4 and 60.7 % of its activity following the 70 °C pre-incubation for 30 s and 1 min, respectively; whereas *R. sphaeriodes* LHS-305 nitrilase only retained 55.3 and 10.1 % of its activity under the same conditions (Fig. 4).

Assessment of NitraS-ATII activity at different NaCl concentrations indicated that the enzyme is not halophilic as its activity decreased with the increase in salt concentration (Fig. 3d).

**Fig. 5:**
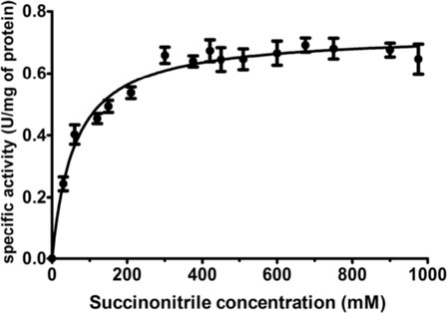
Effect of succinonitrile concentration on NitraS-ATII specific activity. Specific activity was measured in U. One unit (U) of specific activity is defined as 1.0 micromole of ammonia produced in 1.0 min by the enzyme (μmol.min^-1^) under used reaction conditions

The initial velocities of the degradation of succinonitrile catalyzed by NitraS-ATII were determined in a 10 min reaction using different concentrations of succinonitrile ranging from 0 to 975 mmol.L^-1^ under conditions described in the Materials and Methods. NitraS-ATII displayed a typical Michaelis-Menten kinetics based on the obtained initial velocity and specific activity data (Additional file 1: Figure S6 and Fig. 5, respectively). NitraS-ATII *K*_M_ and v_max_ values were determined to be 59.4 ± 6.8 mmol.L^-1^ and 2.432 ± 0.050 μmol NH_3_.min^-1^ (6.081 x 10^-6^ μmol NH_3_.sec^-1^), respectively. Also its specific activity and *k*_cat_ were established as 0.73 U/mg and 0.472 sec^-1^, respectively.

### NitraS-ATII activity maintained at high concentrations of selected metal ions

**Fig. 6:**
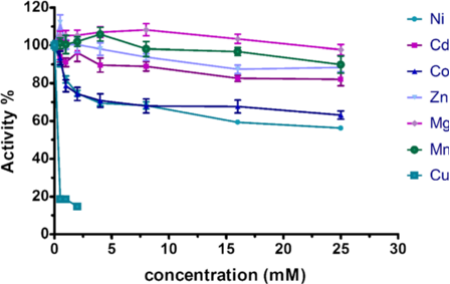
Effect of different metal ions on NitraS-ATII activity. The nitrilase activity is retained at high concentrations of Mg^2+^ and Mn^2+^ and to a lower extent with Zn^2+^. A high degree of tolerance is also observed towards Cd^2+^and Co^2+^, and to a lower extent towards Ni^2+^. Inactivation is observed even with low concentrations of Cu^2+^ and Hg^2+^

The effect of different heavy metals on NitraS-ATII activity is shown in Fig. 6 and Table 1. NitraS-ATII retained near maximal activity even in the presence of high concentrations of ZnCl_2_, MgSO_4_ and MnSO_4_, whereas, a week inhibitory effect was observed upon the addition of NiSO_4_, CdCl_2_ or CoCl_2_. A strong inhibitory effect on NitraS-ATII activity was observed with Cu^2+^ and Hg^2+^ at low concentrations (0.5, 1.0 or 2.0 mmol.L^-1^ CuSO_4_ and 6 or 12 μmol.L^-1^ HgCl_2_). However, in presence of HgCl_2_, the nitrilase activity was nearly reversed when DTT (2.0 mmol.L^−1^) was added to the reaction mixture (97.6 % with 6 μM HgCl_2_ and 90.3 % with 12 μM HgCl_2_). However, this was not the case with CuSO_4_.

**Table 1:**
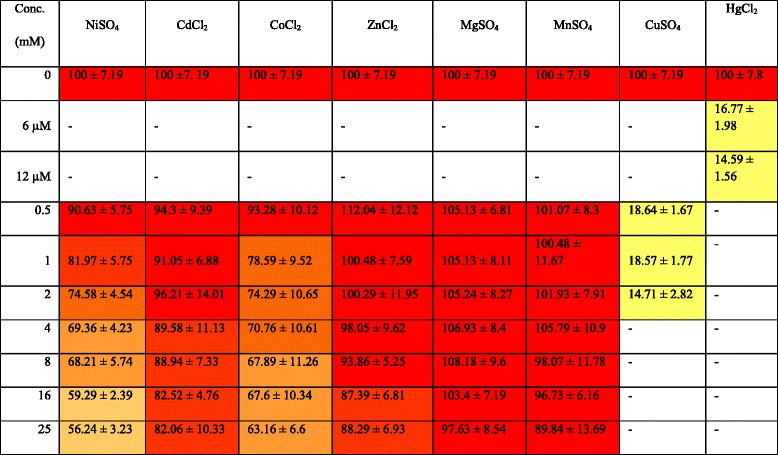
Effect of different metal ions concentrations on the activity of NitraS-ATII. The color code shows the activity percentage, with the highest activity shown in red and the lowest in yellow

**Fig. 7:**
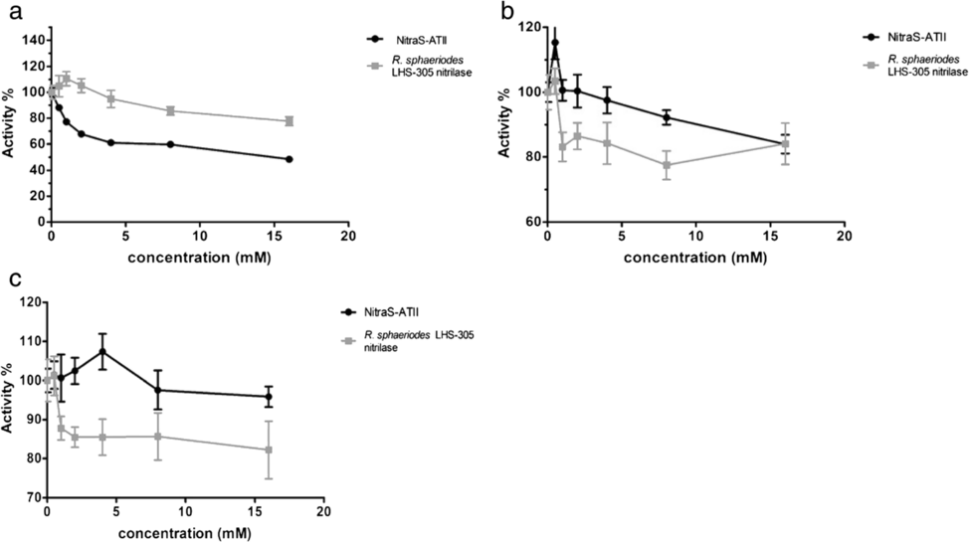
Comparison of the effect of selected metal ions on the activities of NitraS-ATII and *R. sphaeriodes*LHS-305 nitrilase. Each panel shows the activity percentage in the presence of increasing concentrations of a metal ion. **a**. In presence of Ni^2+^, *R. sphaeriodes* LHS-305 nitrilase retains higher activity (*t* test *p*-value = 2.2x10^-3^). **b**. In presence of Zn^2+^, NitraS-ATII retains higher activity (*t* test *p*-value = 9.6x10^-3^). **c**. In presence of Mn^2+^, NitraS-ATII retains higher activity (*t* test *p*-value = 11.8x10^-3^)

Comparison of both NitraS-ATII and *R. sphaeriodes* LHS-305 nitrilase tolerance towards Ni^2+^, Zn^2+^ and Mn^2+^ ions indicated that the inhibitory effect of Ni^2+^ was significantly higher on NitraS-ATII than on the *R. sphaeriodes* enzyme (p–value = 2.2x10^-3^). On the other hand NitraS-ATII showed significantly higher activity in presence of Zn^2+^ (*p*-value = 9.6x10^-3^) and Mn^2+^ (*p*-value = 11.8 x 10^-3^) at concentrations lower than 16 mmol.L^-1^ (Fig. 7).

## Discussion

A considerable attention towards identification and characterization of new nitrilases has increased recently due to their potential uses in green industry and bioremediation. Here, we identified a nitrilase ORF (NitraS-ATII) from the deepest layer (LCL) of the Atlantis II Deep brine pool. The NitraS-ATII ORF was found to be part of a conserved operon, Nit1C, present in several bacterial phyla and in microorganisms that inhabit diverse environments [10]. Different bacterial species belonging to β- and γ-proteobacteria have the same operon arrangement, while in some cyanobacterial species the 7th ORF, a putative FAD-dependent oxidoreductase involved in K^+^ transport, is found on the other end of the cluster on the opposite strand [10]. Despite the fact that the high conservation of this cluster suggests its involvement in a conserved metabolic pathway, the lack of biochemical testing of the genes and the presence of hypothetical proteins with unknown activities hinder the prediction of the function of this operon.

The alignment results of NitraS-ATII ORF and adjacent sequences showed the highest similarity with sequences from *Cupriavidus basilensis* OR16. In addition, other identified ORFs in the LCL metagenomic DNA showed high similarity with ORFs from the same microorganism (data not shown) indicating that the LCL microbial community possess diverse β-proteobacteria close to *C. basillensis*, possibly due to their ability to degrade a wide range of xenobiotics [49].

Testing selected substrates, we found NitraS-ATII to be active towards aliphatic dinitriles, especially succinonitrile. To obtain a complete substrate specificity profile, more substrates should be tested, though. Since some nitrilases were reported to possess amidase activity as well [1], we also tested the activity of NitraS-ATII on acetamide and benzamide. Even though we did not detect any activity using these amides, amidase activity might be present, as a large spectrum of amides would need to be tested to reach a conclusion.

Upon examination of NitraS-ATII activity at different temperatures, we surprisingly found that the optimum temperature range was 35–40 °C. This is in opposition to what would be expected from an enzyme isolated from the ATII LCL environment (temperature of 68 °C). However, pre-incubation of NitraS-ATII at high temperatures for short periods of time did not affect greatly its activity. NitraS-ATII was found to retain more than 60 % of its activity after incubation at 70 °C for 30 s or 1 min. This result could point toward the necessity of additional interactions for NitraS-ATII to maintain its structure and activity under the ATII LCL environmental condition for longer periods.

Since NitraS-ATII was found to not be halophilic, losing more than 75 % of its activity at NaCl concentrations higher than 2.0 mol.L^-1^, it would be expected that the microorganism from which the gene was isolated would produce osmolytes to survive the high salinity of the ATII LCL environment (250 parts per thousand). Osmolytes have been known to have protecting roles against other stresses rather than osmolarity [50]. Even though a consensus about the in vivo relevance of osmolytes to thermostability of proteins have not been reached, some halophilic (hyper) thermophiles accumulate them in growth conditions with supraoptimal temperature, denoting a possible auxiliary role [51]. On the other hand, protein-protein interactions involved in re-folding of denatured proteins could play a rather important role in the stabilization of NitraS-ATII, even if the microorganism accumulated osmolytes. *Methanococcus jannaschii* glutamine synthetase was not significantly stabilized by osmolytes at the normal growth temperature of the organism (85 °C), even though loss of *M. jannaschii* aspartate transcarbamoylase activity was prevented by such molecules under the same condition [52].

Comparing their short-period thermal stability profile, NitraS-ATII was found to retain most of its activity at 70 °C, while the activity of *R. sphaeriodes* LHS-305 nitrilase dropped significantly after being subjected to the same conditions. The difference in expected number of salt bridges between the two enzymes – NitraS-ATII was predicted to have 119 compared to 102 in case of *R. sphaeriodes* LHS-305 nitrilase – could be high enough to account for the observed higher thermal stability of NitraS-ATII. An increased number of salt bridges has been considered one of the characteristics responsible for the enhancement of protein thermostability [53–56]. Recently, a mesophilic β-glucosidase from *Bacillus polymyxa* was engineered to become thermostable by the creation of salt bridges at positions inferred as suitable by computational analysis of its three-dimensional structure. Addition of 4 new salt bridges at specific positions were sufficient to increase the melting temperature (T_m_) in 15.7 °C [57]. Moreover, alignment of NitraS-ATII with *R. sphaeriodes* LHS-305 nitrilase showed 4 glycine to alanine substitutions in NitraS-ATII, which may contribute to its higher thermal stability as well.

Studies on the Atlantis II Deep brine pool and particularly the lowest and most isolated part of the pool, the LCL, revealed its high heavy metal content [58]. Thus, it was expected that NitraS-ATII might show some tolerance to certain metals. Although some studies have shown the effect of metals on different nitrilases, they were confined to studying the effect of low concentrations of metal ions (1.0 or 5.0 mmol.L^-1^) [7, 17, 59, 60], while here we studied the effect of several metals at concentrations up to 25 mmol.L^-1^. In general, NitraS-ATII showed high tolerance towards most of the tested metal ions. Nevertheless, strong inhibitory effects of Cu^2+^ and Hg^2+^ ions were observed, which could be explained by the possible complex formation between these ions and the thiol group in the cysteine residue of the catalytic triad of the enzyme. Reversal of nitrilase activity by the addition of DTT in presence of Hg^2+^, but no in presence of Cu^2+^, reflected the different concentrations of metal ions present, about a hundred times lower for Hg^2+^. Moreover, possible reduction of Hg^2+^ by DTT and the consequent removal of the metal (Hg^0^) from the reaction mixture by evaporation could diminish more the concentration of this cation.

Upon comparing the results of metal tolerance of NitraS-ATII with those of *R. sphaeriodes* LHS-305 nitrilase, NitraS-ATII showed significantly higher tolerance to high concentrations of Mn^2+^ and Zn^2+^, which are both present at high concentrations in the LCL [58]. On the other hand, *R. sphaeriodes* LHS-305 nitrilase showed higher tolerance towards Ni^2+^. As no nickel was detected in the LCL, it seems that the inhibitory effect of nickel on NitraS-ATII has no adverse consequences in its natural environment. It also indicates a molecular evolution of the enzyme to gain tolerance specifically towards the metal ions present in the LCL environment.

## Conclusion

Identification of new nitrilases is of utmost importance due to the promises they hold in several biotechnological and pharmaceutical applications. Mining available metagenomic datasets, particularly of those from extreme environments, provides an opportunity for the identification of several extremophilic enzymes with diverse applications. In this study, a nitrilase (NitraS-ATII) gene was identified, sequenced and cloned followed by expression and purification of the protein. Functional studies revealed that the enzyme is active towards aliphatic dinitriles. Such a characteristic could be further exploited in the synthesis of cyanocarboxylic acids. NitraS-ATII showed higher thermal stability compared to a closely related nitrilase. Furthermore, it displayed high tolerance to different metals. Further studies on NitraS-ATII are needed to recognize its stereo- and regio-selectivity and identify the number of subunits of the active protein. Moreover, additional studies to biochemically characterize the proteins in the operon of NitraS-ATII would be of interest to unveil the functional purpose of the widespread and conserved Nit1C gene cluster. This study, therefore, provides an initial platform to assess the biotechnological potential of this newly identified nitrilase.

## Availability of supporting data

GenBank accession number of NitraS-ATII gene: KT354778.

## Additional file

Additional file 1: Figure S1. Artemis visualization of NitraS-ATII ORF and surrounding ORFs with positions of primers used in this work. **Figure S2**. Nit1C Operon present in contig00026 of the Atlantis II Deep LCL metagenomic assembly. A. **Figure S3**. DNA sequence of NitraS-ATII gene within its genomic context and showing its regulatory elements. **Figure S4**. Multiple sequence alignment of NitraS-ATII with different nitrilases. **Figure S5**. Qualitative detection of nitrilase activity. **Figure S6**. Effect of succinonitrile concentration on the initial velocity of NitraS-ATII. **Table S1**. List of assembled contigs with the Carbon-Nitrogen hydrolase functional domain. **Table S2**. List of used primers, annealing temperatures and amplicon sizes. **Table S3**. Position and annotation of genetic elements in the Nit1C operon within contig00026 of the Atlantis II Deep LCL metagenomic assembly. (DOCX 3860 kb)

## Abbreviations

ATII: Atlantis II Deep
BSA: Bovine serum albumin
CDS: coding DNA sequence
CTD: conductivity, temperature and depth
DTT: Dithiothreitol
FAD: Flavin adenine dinucleotide
IPTG: Isopropyl β-D-1-thiogalactopyranoside
LB: Luria bertani
LCL: Lower convective layer
MW: molecular weight
OD_600_: Optical density at 600 nm
ORF: open reading frame
PDB: Protein data bank
PMSF: Phenylmethylesulfonyl fluoride
SDS-PAGE: Sodium dodecyl sulfate-polyacrylamide gel electrophoresis
X-gal: 5-bromo-4-chloro-3-indolyl-β-D-galactopyranoside
