# Supplementary material for: Red Sea Atlantis II brine pool nitrilase with unique thermostability profile and heavy metal tolerance
Source: BMC Biotechnol. 2016 Feb 11;16:14. doi: 10.1186/s12896-016-0244-2 (PMC4751646; doi:10.1186/s12896-016-0244-2)
Supplement: Additional file 1: Figure S1. — Artemis visualization of NitraS-ATII ORF and surrounding ORFs with positions of primers used in this work. Figure S2. Nit1C Operon present in contig00026 of the Atlantis II Deep LCL metagenomic assembly. A. Figure S3. DNA sequence of NitraS-ATII gene within its genomic context and showing its regulatory elements. Figure S4. Multiple sequence alignment of NitraS-ATII with different nitrilases. Figure S5. Qualitative detection of nitrilase activity. Figure S6. Effect of succinonitrile concentration on the initial velocity of NitraS-ATII. Table S1. List of assembled contigs with the Carbon-Nitrogen hydrolase functional domain. Table S2. List of used primers, annealing temperatures and amplicon sizes. Table S3. Position and annotation of genetic elements in the Nit1C operon within contig00026 of the Atlantis II Deep LCL metagenomic assembly. (DOCX 3860 kb) [file 12896_2016_244_MOESM1_ESM.docx]

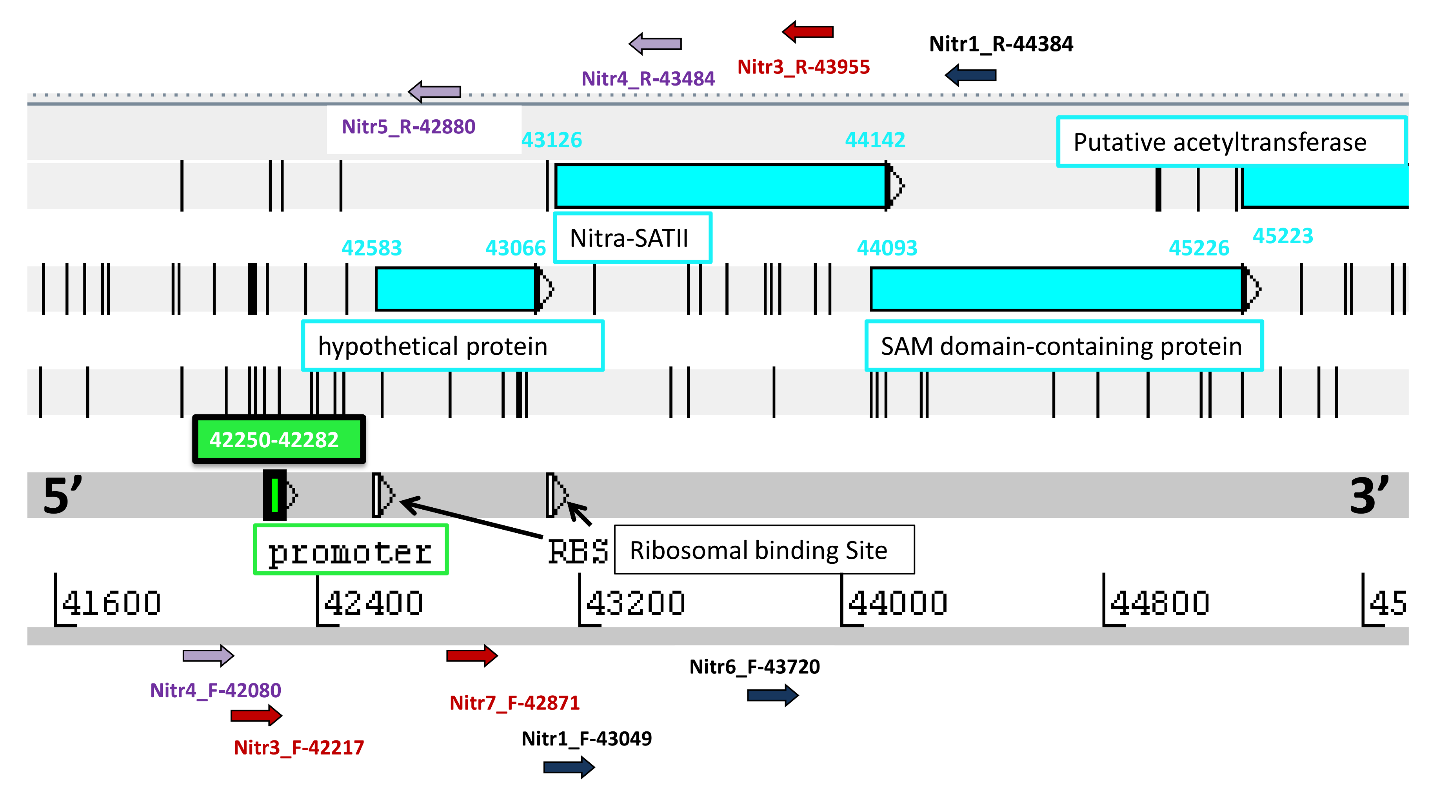


Figure S1. Artemis visualization of NitraS-ATII ORF and surrounding ORFs with positions of primers used in this work. Schematic arrows with numbers show the positions of the first base of each primer. Positions are in reference to the assembled contig (contig00026). Cyan rectangles represent NitraS-ATII ORF and surrounding ORFs. Annotation of putative proteins are indicated below, or above, the depicted ORFs. Promoter region is shown in green with its corresponding position.

**
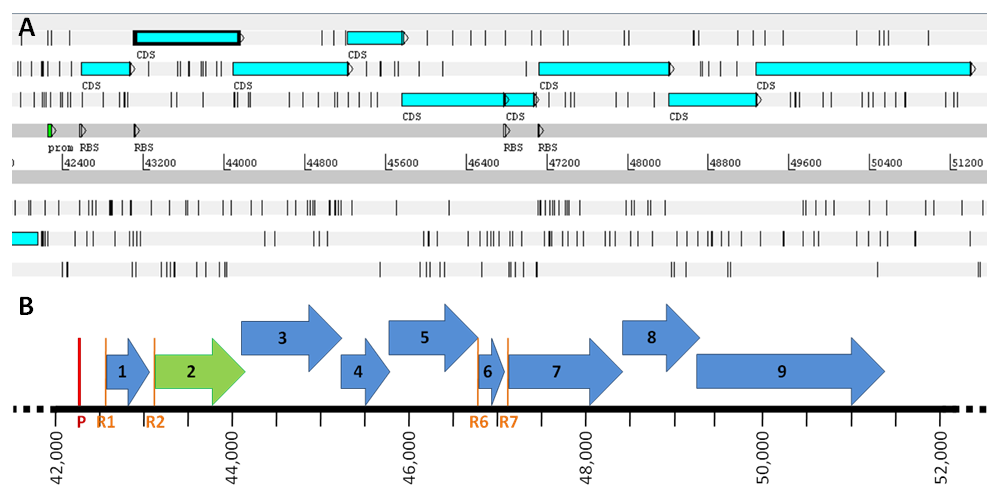
**

Figure S2. Nit1C Operon present in contig00026 of the Atlantis II Deep LCL metagenomic assembly. A. Artemis visualization of the ORFs in the Nit1C operon where the NitraS-ATII gene is present (second ORF, bold border). ORFs are shown as cyan arrows at different reading frames in the forward strand. The promoter region is indicated with a small green arrow starting at 42,250 bp, while NitraS-ATII CDS extends from 43,126 to 44,142 bp. B. Schematic view of the Nit1C operon, ranging from 42,250 to 51,394 bp. Detailed information about operon elements are present in supplementary table S3. Numbered ORFs were annotated as follows: 1, conserved hypothetical protein; 2, NitraS-ATII nitrilase (biochemically characterized in this paper, in green); 3, putative radical SAM domain-containing protein; 4, putative acetyltransferase; 5, selenophosphate synthetase-related protein; 6, conserved hypothetical protein; 7, putative FAD-dependent oxidoreductase with predicted K^+^ transport function; 8, conserved hypothetical protein; 9, putative methylmalonyl-CoA mutase. P, operon promoter; R1, R2, R6 and R7, ribosomal binding sites for ORFs 1, 2, 6 and 7, respectively.


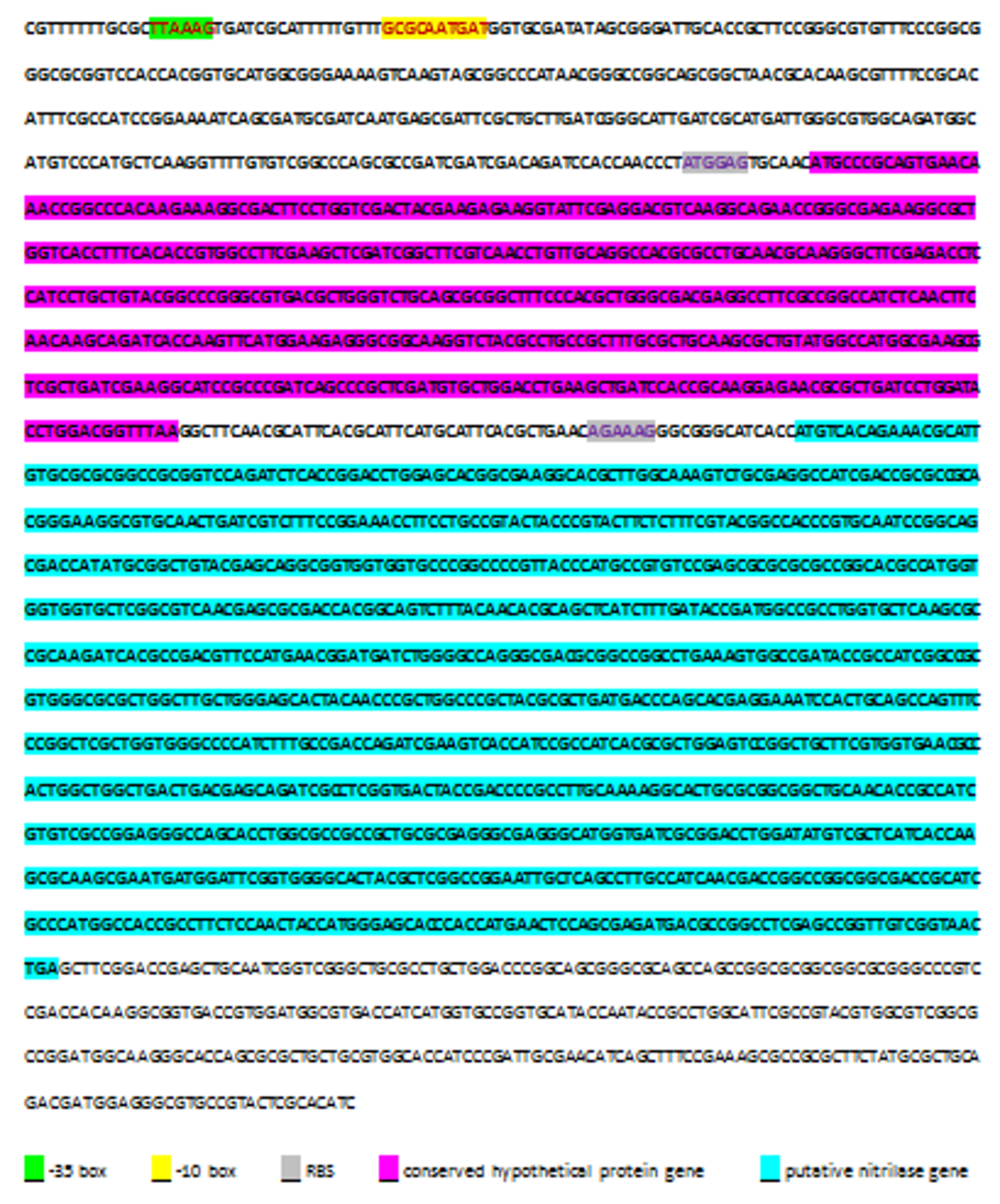


Figure S3. DNA sequence of NitraS-ATII gene within its genomic context and showing its regulatory elements. The conserved hypothetical protein and NitraS-ATII ORFs were identified by means of analysis of the consensus sequence of contig00026 by MetaGeneAnnotator. Putative function was annotated based on BLASTp results against NCBI nr database. Regulatory and promoter elements were identified using bProm (SoftBerry®). Ribosomal binding sites were manually annotated.


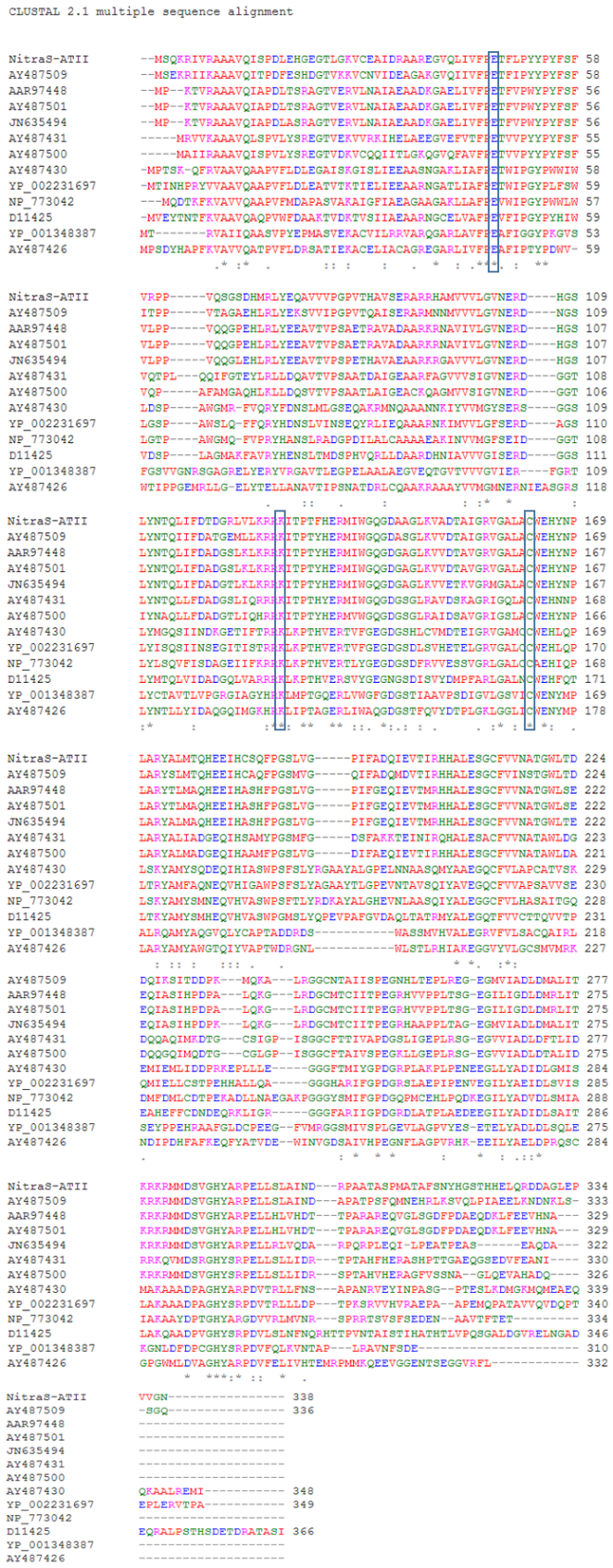


Figure S4. Multiple sequence alignment of NitraS-ATII with different nitrilases. Close nitrilase homologs of NitraS-ATII (first sequence), identified by their NCBI accession numbers, were selected based on their high similarity and, subsequently, subjected to multiple sequence alignment using ClustalW2. Residues of the catalytic triad are shown in rectangles.


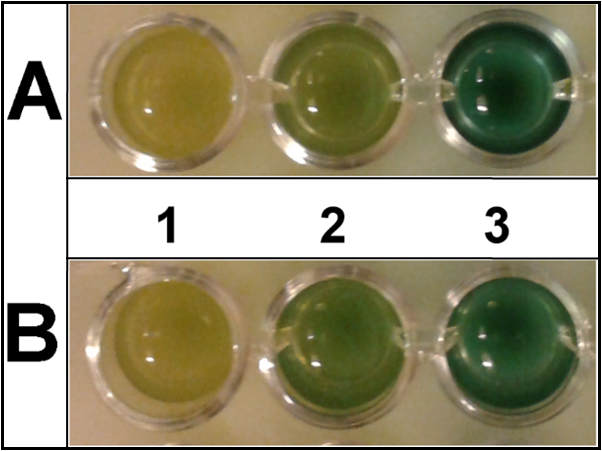


**Figure S5. Qualitative detection of nitrilase activity.** Production of ammonia was detected by colorimetric changes of the bromothymol indicator due to the increase in pH, following the procedure described in Materials and Methods. Different cultures of *E. coli* DL21 (DE3) were subjected to the assay: induced (1) and uninduced (2) p‑NitraS‑ATII transformed cells; and untransformed cells (3). Results for succinonitrile (A) and glutaronitrile (B) as substrates are presented.


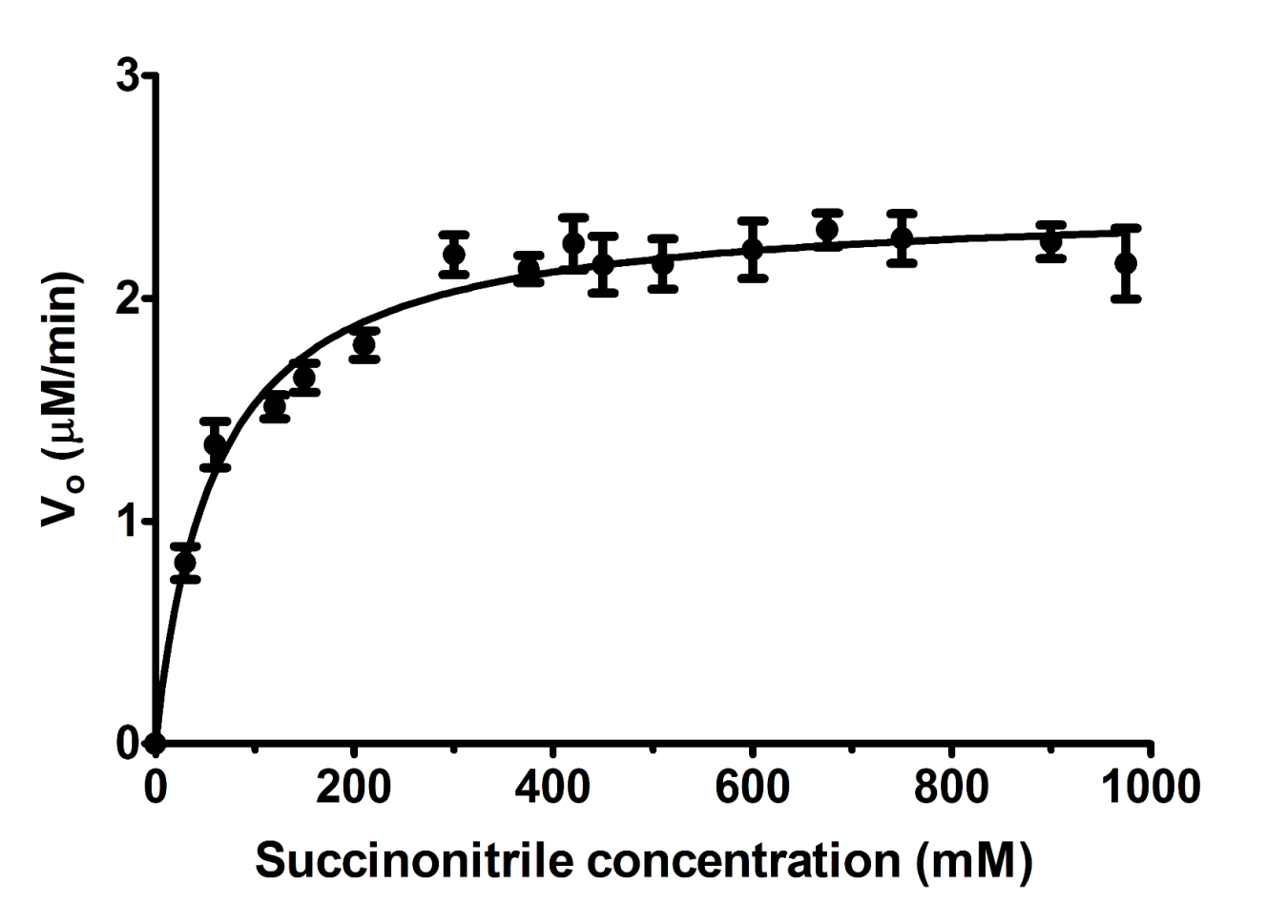


**Figure S6. Effect of succinonitrile concentration on the initial velocity of NitraS-ATII.** Employing the quantitative procedure described on Materials and Methods, initial velocities were determined in 10-min reactions at different concentrations of substrate. Obtained data indicated that NitraS-ATII followed Michaelis-Menten kinetics, displaying K_M_ and v_max_ values of 59.4 ± 6.8 mmol.L^‑1^ and 2.432 ± 0.050 µmol NH_3_.min^‑1^, respectively. Succinonitrile was chosen as preferred substrate based on results obtained by the qualitative assay. Measurements were done in triplicate and data is presented as mean ± SE. The graph was plotted and the kinetics parameters were determined using GraphPadPrism®.

Table S1. List of assembled contigs with the Carbon-Nitrogen hydrolase functional domain. Number of reads used in the assembly and length of the contig are presented. Contig 15 was selected for further analysis and used to identify a genomic region containing the NitraS-ATII CDS in the Atlantis II Deep LCL metagenomic assembly.

| **Contig** | **Length (bp)** | **Number of reads** |
| --- | --- | --- |
| Contig1 | 615 | 2 |
| Contig2 | 489 | 2 |
| Contig3 | 573 | 2 |
| Contig4 | 480 | 2 |
| Contig5 | 734 | 2 |
| Contig6 | 540 | 3 |
| Contig7 | 906 | 4 |
| Contig8 | 866 | 7 |
| Contig9 | 760 | 8 |
| Contig10 | 982 | 8 |
| Contig11 | 1037 | 12 |
| Contig12 | 1036 | 21 |
| Contig13 | 1042 | 28 |
| Contig14 | 955 | 30 |
| Contig15 | 1209 | 40 |

Table S2. List of used primers, annealing temperatures and amplicon sizes.

| **Primer** | **Sequence** | **Annealing temperature T_a_ (°C)** | **Amplicon size (bp)** | **Description of the amplified sequence** |
| --- | --- | --- | --- | --- |
| Nitr1_F  Nitr1_R | GATACCTGGACGGTTTAAGG  GAAAGCTGATGTTCGCAAT | 54 | 1336 | NitraS-ATII ORF plus 76 bp upstream and 243 bp downstream |
| Nitr6_F  Nitr1_R | CAGATCGAAGTCACCATCC  GAAAGCTGATGTTCGCAAT | 54 | 665 | Part of the NitraS-ATII ORF plus 243 bp downstream |
| Nitr3_F  Nitr3_R | GTCTCAGGCTACCACAATGA  GTGATGAGCGACATATCCAG | 54 | 1739 | Operon promoter region, first ORF in the operon and NitraS-ATII ORF minus 188bp in the 3’ end |
| Nitr3_F  Nitr4_R | GTCTCAGGCTACCACAATGA  TCGGTATCAAAGATGAGCTG | 54 | 1268 | Operon promoter region, first ORF in the operon and 359 bp of the 5’end of NitraS-ATII ORF |
| Nitr7_F  Nitr3_R | CAACAAGCAGATCACCAAGT  GTGATGAGCGACATATCCAG | 54 | 1085 | 3’end of the first ORF in the operon plus the 5’end of the NitraS-ATII ORF |
| Nitr4_F  Nitr4_R | ATCAGGTAAGCGGGTTTG  TCGGTATCAAAGATGAGCTG | 54 | 1405 | Upstream the promoter region to the 5’end of the NitraS-ATII ORF |
| Nitr4_F  Nitr5_R | ATCAGGTAAGCGGGTTTG  CTGCTTGTTGAAGTTGAGATG | 54 | 801 | Upstream the promoter region to the 5’end of the first ORF in the operon |

**Table S3. Position and annotation of genetic elements in the Nit1C operon within contig00026 of the Atlantis II Deep LCL metagenomic assembly.** Codes prior to elements’ definitions are related to the scheme of the Nit1C operon presented in supplementary figure S2B.

| **ORFs and *cis*-elements** | **Position in Contig00026 (length of the element)** | **Notes** |
| --- | --- | --- |
| (**P**) Promoter:  -10 box CGCAATGAT  -35 box TTAAAG  Known TF binding sites:  purR CGTTTTTT  rpoD15 TTTTGTTT | 42,250 … 42,282 (32 bp) | Detection by Softberry® bprom.  Promoter LDF 1.94  -10 box Score 32  -35 box Score 31  purR Score 8  rpoD15 Score 9 |
| (**R1**) RBS ATGGAG | 42,572 … 42,577 (5 bp) | Manually detected. |
| (**ORF1**) Conserved hypothetical protein | 42,584 … 43,066 (482 bp) | † |
| (**R2**) RBS AGAAAG | 43,107 … 43,112 (5 bp) | Manually detected. |
| (**ORF2**) **NitraS-ATII nitrilase** | 43,126 … 44,142 (1,016 bp) | † |
| (**ORF3**) Putative radical SAM domain-containing protein | 44,093 … 45,226 (1,133 bp) | † |
| (**ORF4**) Putative acetyltransferase | 45,223 … 45,768 (545 bp) | † |
| (**ORF5**) Selenophosphate synthetase-related protein | 45,765 … 46,775 (1,010 bp) | † |
| (**R6**) RBS AGGAGA | 46,775 … 46,780 (5 bp) | Manually detected. |
| (**ORF6**) Conserved hypothetical protein | 46,785 … 47,072 (287 bp) | † |
| (**R7**) RBS AGGAAG | 47,112 … 47,117 (5 bp) | Manually detected. |
| (**ORF7**) Putative FAD-dependent oxidoreductase with predicted K^+^ transport function | 47,126 … 48,409 (1,283 bp) | † |
| (**ORF8**) Conserved hypothetical protein | 48,411 … 49,274 (863 bp) | † |
| (**ORF9**) Putative methylmalonyl-CoA mutase | 49,271 … 51,394 (2,123 bp) | † |

† Detected by MetaGeneAnnotator and annotated by BLASTp search against NCBI nr.
